# Molecular characterization of *Escherichia coli* isolate from the Greys-Hoback Watershed, Wyoming

**DOI:** 10.1128/mra.01187-24

**Published:** 2025-07-31

**Authors:** Anahita Ghorbani Tajani, Aniket Sharma, Kelsey Ruehling, Sarah Collins, Bledar Bisha

**Affiliations:** 1 Department of Animal Science, University of Wyoming 4416, Laramie, Wyoming, USA; 2 Department of Zoology and Physiology, University of Wyoming 4416, Laramie, Wyoming, USA; Indiana University Bloomington, Bloomington, Indiana, USA

**Keywords:** antimicrobial resistance, Wyoming water quality, *Escherichia coli*, *bla*_TEM-150_, genome sequencing

## Abstract

This study investigates antimicrobial resistance and virulence profiles in *Escherichia coli* isolates from four streams in the Greys-Hoback watershed, Wyoming, USA. Using high-throughput DNA sequencing, we identified spatial and temporal variation, providing valuable data for addressing water quality issues and informing local water management strategies.

## ANNOUNCEMENT

The Greys-Hoback watershed in Wyoming, including Fish and Flat Creeks, has recently experienced elevated levels of *Escherichia coli* (*E. coli*) that sometimes exceed recreational use standards for water quality (1). This study was conducted to detail the spatial distribution of *E. coli* and to uncover any antimicrobial resistance and virulence traits prevalent in the isolates. In 2021, surface water samples were collected at 43.49565°N, 110.871933°W, 6 inches below the surface using sterile bottles, stored on ice (~4°C), and transported to the lab within 24 h. Field collections of water samples followed stringent protocols to minimize contamination.

Upon sample receipt, *E. coli* enumeration followed the EPA Method 1603 (*n* = 263) (2), utilizing membrane filtration and modified mTEC agar. Isolate confirmation as *E. coli* (*n* = 218) was performed via matrix-assisted laser desorption/ionization-time of flight mass spectrometry in the form of a Bruker Microflex LRF instrument with Biotyper-v. 3.0 software.

A minimum inhibitory concentration (MIC) assay followed Clinical and Laboratry Standard Institute guidelines (3), where a standardized *E. coli* inoculum was exposed to 14-precoated antibiotic microtiter plate (CMV3GANF), incubated at 37°C for 16–20 h, and the MIC was determined using SWIN software-v.3.3 (ThermoFisher Scientific). The multi-drug-resistant isolates (*n* = 30) were grown on Brain Heart Infusion agar at 37°C for 18 h. These isolates underwent DNA extraction using the DNeasy PowerLyzer Microbial Kit (Qiagen), and library preparation used the NEBNext Ultra DNA Library Prep Kit for Illumina (NEB, USA) following standard protocols. In brief, the PCR amplification included five cycles for simultaneous indexing and amplification, followed by size selection cleanup. DNA samples were sonicated to 350 bp, end-polished, A-tailed, ligated with adaptors, and PCR amplified. Library size was analyzed using Agilent 2100 Bioanalyzer and quantified via real-time PCR.

High-throughput sequencing for each isolate was achieved using the NovaSeq 6000 platform (4) (150 bp paired-end reads, 500 ng genomic DNA/sample). Sequencing achieved a coverage of 100× per sample. Sequencing reads were quality-checked with FastQC-version 0.12.0 (5), trimmed with Trimmomatic-version 0.36 (6), and assembled using SPAdes-version 3.15.5 (7). Genome annotation was performed with PGAP-version 6.8 (8), antibiotic resistance genes (ARGs) were analyzed with ABRicate-version 0.2-0 (9), virulence genes with VirulenceFinder-version 2.0 (10), and plasmids with PlasmidFinder-version 2.0.1 (11). Default parameters were used except where otherwise noted. Here, we present the genome of isolate S5, selected for its diverse ARGs, including *bla*_EC-18_, *floR*, *tet(A), aph(6)-Id*, *aph(3'')-Ib*, *sul2*, and *bla*_TEM-150_ were predicted and identified in the strain S5 (*n* = 1). Plasmid analysis revealed the presence of IncFIA, IncFIB(AP001918), and IncFIC(FII). Virulence factors predicted included *anr*, *csgA, fdeC*, *fimH*, *gad*, *hlyE*, *iss*, *lpfA*, *nlpI*, *ompT*, *terC*, *traJ*, *traT*, *yehA*, *yehB*, and *yehC*. The genome analysis revealed a GC content of 50%, with 4,511,195 total reads. The assembly comprised 85 contigs, with an *N*_50_ of 183,443 and an *L*_50_ of 10. The assembled genome achieved a completion rate of 90.22%, and a total of 4,738 genes were predicted in the assembled genome based on annotation performed using PGAP-version 6.8. These findings present significant implications for public health and local water management strategies.

## Data Availability

The sequencing data and genome assembly have been deposited with accession number BioSample SAMN43082930. This Whole Genome Shotgun project has been deposited in GenBank under the accession number JBIEAH010000000 and Sequence Read Archive (SRA) PRJNA1146023, SRR30993544 accessible through NCBI.
